# Static allometries of caste-associated traits vary with genotype but not environment in the clonal raider ant

**DOI:** 10.1073/pnas.2501716122

**Published:** 2025-07-22

**Authors:** Patrick K. Piekarski, Stephany Valdés-Rodríguez, Waring Trible, Daniel J. C. Kronauer

**Affiliations:** ^a^Laboratory of Social Evolution and Behavior, The Rockefeller University, New York, NY 10065; ^b^Howard Hughes Medical Institute, The Rockefeller University, New York, NY 10065; ^c^John Harvard Distinguished Science Fellowship Program, Harvard University, Cambridge, MA 02138

**Keywords:** allometric plasticity, caste development, developmental plasticity, polyphenism, systemic growth

## Abstract

Polyphenic traits in animals often exhibit nonlinear scaling with body size. Static allometries (i.e., scaling relationships) themselves can exhibit plasticity, such that individuals of the same size and genotype differ in body proportions across different environments. In ants, both larval environment and genotype regulate the expression of caste-associated traits, including body size and ovariole number. However, it remains untested whether caste-associated traits are independently regulated by environmental variables or whether they covary due to coupled developmental mechanisms. If caste traits are regulated independently, developmental plasticity should affect both trait expression and the scaling relationships between traits. Using the clonal raider ant, *Ooceraea biroi*, we tested this by manipulating the rearing environment of genetically identical larvae. We found that caregiver genotype, temperature, and food quantity influenced caste morphology strictly in tandem with body size, producing similar static allometries across rearing conditions (i.e., no allometric plasticity was detected). In contrast, clonal genotypes differed in average body size and their static allometries. Thus, size-matched individuals of the same genotype from different rearing environments exhibited no differences in mean caste trait expression, while those of different genotypes did. This absence of plasticity in the static allometries of different caste traits suggests that they are developmentally coupled due to systemic regulatory factors. Our findings contrast with reports of allometric plasticity in other insects, suggesting that ant caste traits are exceptionally integrated and therefore constrained in their independent responses to environmental variation. We discuss how these results inform contemporary hypotheses for ant caste development and evolution.

## Significance

How an individual’s phenotype arises from interactions between its genotype and the rearing environment is an important question in biology. Female ants are a powerful system to study this question because they can develop into divergent types, or castes, such as workers and queens. Here, we show that environmental effects on the caste morphology of individuals are coupled with effects on body size and fail to find evidence for size-independent regulation of caste morphology. Genetic differences, on the other hand, affected both body size and its relationship with caste phenotypes, implying that genotype can influence adult caste morphology either by altering body size or the allometric relationship between body size and caste trait expression.

The environment experienced during development can profoundly influence the phenotype of an individual. When a genotype generates different phenotypes (i.e., trait values) in response to environmental cues and/or signals, this is referred to as developmental plasticity (1). Developmental plasticity can be visualized using a reaction norm that shows how the trait expression of a single genotype varies across a range of environmental conditions. The most striking manifestations of developmental plasticity are found in polyphenisms, for which there are two or more distinct adaptive phenotypes (2, 3). Among the most remarkable examples of insect polyphenisms are the anatomical and morphological differences between alternative male morphs in dung beetles and between the female castes of social insects.

In most ants, winged queens specialize in egg laying, while wingless workers specialize in rearing the brood and defending the nest (4). Compared to queens, workers have ovaries that are reduced or absent (5), reduced development of eyes and ocelli (6, 7), and no wings (7, 8). Within a species, variation in these physical adult traits is typically due to developmental plasticity and their expression is correlated: The expression of one caste-associated trait is generally predictive of all other traits and, therefore, the caste identity of the individual (9). Among ants, workers are generally smaller than queens, and it has been suggested that the differential growth of wings and other tissues is tightly correlated with the body size of developing larvae (10–12). In this study, we investigate how genetic and environmental variation affects this pervasive correlation between ant caste phenotypes and body size.

Polyphenic traits are often defined by nonlinear scaling relationships (i.e., nonlinear static allometries) between trait expression and body size (3, 10, 13–16). Static allometry describes the scaling relationship between two traits among conspecific individuals measured at the same stage of development (17). In this study, our usage of the term static allometry refers specifically to the scaling relationship between the expression of a focal caste trait and body size for a group of genetically identical individuals. Nonlinear static allometries often follow discontinuous or sigmoidal functions, reducing the frequency of intermediate states and potentially improving the adaptive function of the polyphenism (14, 18–20). For a size-based polyphenism, as in dung beetles and ants, the midpoint between alternative phenotypes is often defined as the body size threshold (15, 19, 21). For polyphenic traits that are strongly associated with adult body size, static trait allometries can be viewed as reaction norms with body size as the environmental x-axis (14). Although body size is highly predictive of trait expression in these systems, this does not imply that body size itself is the causal factor.

Queen pheromones (22), wounding (23), nutrition (24, 25), temperature (26), caregiver genotype (27, 28), colony size (29), and maternal effects (30, 31) are all known to affect caste development in ants. Beyond developmental plasticity, caste development can also be affected by genetic variation, such as when certain genotypes have an increased probability for queen development. These genetic biases can range from minor to absolute (32, 33). For example, in polyandrous species, where queens mate with several males, patrilines can be differentially biased toward worker and/or queen development (34, 35), as well as differ in their propensities to develop into different worker subcastes (36).

Genotypic differences in the static allometry of caste traits in ants are well known and likely ubiquitous (9, 34, 37–39). Studies of developmental plasticity from other animals with size-based polyphenisms have also revealed modest differences in the static allometry of polyphenic traits due to the environment, a phenomenon called allometric plasticity (40). If the static allometry relating a trait with body size is itself plastic, this can cause individuals of the same size and genotype to differ in trait expression. For instance, environmental variables might affect the expression of a particular trait without corresponding changes in body size or other traits. However, to our knowledge, no research has addressed whether developmental plasticity in ants can affect caste trait allometries or whether genetically encoded static allometries are invariant across different environments (10–12, 41). To date, investigations into allometric plasticity for polyphenic traits in insects have primarily focused on the effects of diet on the static allometries of continuous characters, such as male secondary sexual traits (40, 42, 43). More recently, however, investigators found that food quality induced allometric plasticity for a discrete secondary sexual trait in male mites (44), and another revealed that the relationship between male dung beetle horn length and body size can be altered by temperature (45).

In holometabolous insects, adult structures develop from clusters of cells called imaginal primordia or discs (46, 47). As larvae grow, these discs expand through cell proliferation (47). At metamorphosis, the cells of the imaginal discs rapidly differentiate and evaginate to form adult structures like legs, eyes, and wings (46). Organization of the adult reproductive system, including ovarioles in Diptera (48, 49) and spermathecae in Diptera and Hymenoptera (50), begins during late larval development and continues through the early pupal stage. Environmental variables may either regulate the growth and differentiation of different imaginal tissues independently of each other and overall body size (i.e., modular tissue growth), or in a coordinated, systemic manner. For caste-associated traits in ants, it remains unclear which of these scenarios best describes the developmental regulation of tissue growth and body size.

**Fig. 1.**
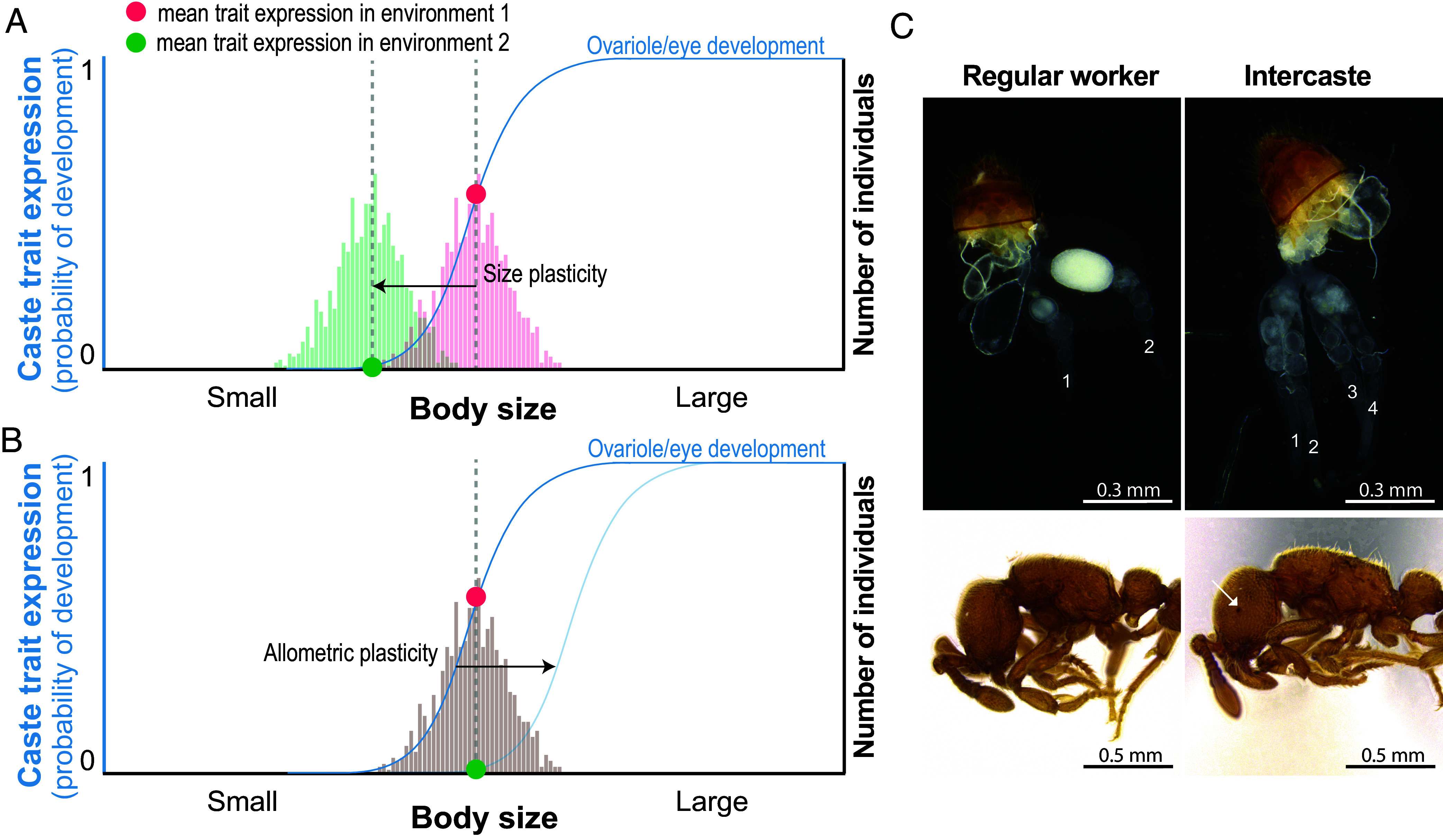
Developmental plasticity in caste morphology may be coupled to body size plasticity and/or be decoupled via allometric plasticity. (*A* and *B*) The static allometry, representing the relationship between body size and a caste-associated trait, such as ovariole and vestigial eye development, is shown as a blue sigmoidal function. For continuous traits, the sigmoid represents the expected mean trait value as a function of body size, and for discrete traits, it represents the probability of developing either state as a function of body size. Body size distributions from two different rearing environments are represented with color-coded histograms. Red and green circles represent caste trait expression, in this case the probability of developing intercaste morphology, in the same hypothetical average individual in two different environments. (*A*) Environmental effects on caste morphology are correlated with changes in body size. In this case, decreased body size and reduced probability of developing intercaste morphology arise in tandem. Here, developmental plasticity in caste morphology is coupled with body size plasticity. (*B*) Alternatively, developmental plasticity in caste morphology is due to allometric plasticity, such that environmentally induced changes in caste trait expression are not coupled or proportional with the expected changes in body size. Here, the static allometries have shifted so that expression of intercaste morphology now requires larger body sizes. In the presence of allometric plasticity, size-matched individuals of the same genotype from different environments differ in caste morphology on average, implying that some factors independently regulate caste trait expression and body size. (*C*) Caste-associated morphologies of *Ooceraea biroi* regular workers (*Left* column) and intercastes (*Right* column). Regular workers have two ovarioles and lack vestigial eyes, while intercastes, defined by having four or more ovarioles, typically have vestigial eyes. The white arrow denotes vestigial eye.

If the developmental plasticity of caste traits is strongly coupled, allometric plasticity should be limited or absent. In this scenario, the static allometries relating body size and caste phenotypes should be insensitive to environmental perturbation: Even if individuals from different environments differ in average size and caste trait expression, size-matched individuals will have identical phenotypes on average (Fig. 1*A*). In contrast, environmental factors could affect caste traits without the expected corresponding effects on body size or other caste traits, causing size-matched individuals of the same genotype to differ in mean caste trait expression across different rearing environments, thereby revealing allometric plasticity (Fig. 1*B*). This distinction is important for our understanding of caste evolution because allometric plasticity has the potential to tremendously increase the spectrum of phenotypes available to each genotype. Alternatively, allometric plasticity may be limited or absent due to developmental coupling of caste traits. If caste traits and body size remain correlated in the same way across environmental contexts, this could greatly simplify future analyses of caste phenotypes by allowing us to ignore the specific environmental influences that affected the size of an individual and instead analyze caste traits using a modified reaction norm with body size on the x-axis and any number of caste traits on the y-axes (10–12, 14). Distinguishing these alternatives is thus important both to address a major empirical question and to identify analytical tools for future research.

Here, we use the clonal raider ant, *Ooceraea biroi*, to test whether developmental plasticity of caste-associated traits is strictly coupled with body size plasticity (Fig. 1*A*) or whether the static allometries of these traits exhibit plasticity (Fig. 1*B*). We also test whether different clonal genotypes, A, B, and M, which are closely related and ultimately derived from Bangladesh (51, 52), exhibit differences in static allometry. *O. biroi* is clonal and reproduces via phasic colony cycles, in which individuals develop in tightly synchronized age cohorts. This species therefore affords maximal experimental control over genetic and environmental effects and provides a unique opportunity to compare static allometries across multiple genotypes and environments. Although fully formed morphological queens are absent in *O. biroi*, a subset of individuals, called intercastes, have increased body size and partially queen-like traits. Unlike regular workers, which have two ovarioles, intercastes have four or more ovarioles, and the presence of additional ovarioles is associated with the formation of vestigial eyes (Fig. 1*C*), an increase in mesosomal segmentation reflective of incipient wing development (9, 53), as well as reduced foraging activity (54). The workers of *O. biroi* are phenotypically homologous to the workers of related *Ooceraea* species with morphologically distinct queens (9). That individuals larger than regular workers can also show increased ovariole numbers, eye development, mesosomal segmentation, and wing development is not unique to *O. biroi*, but a phenomenon observed in all ants for which data are available (10). These observations indicate that the worker-intercaste distinction exhibited by *O. biroi* is reflective of similar associations between caste traits and body size that are widespread in ants, making this species an ideal model to test for allometric plasticity in ant caste development.

## Results

### Environmental Effects on Caste Morphology.

Prior experiments showed that the frequency of intercastes plastically varies between larval cohorts due to both the genotype of the caregiver workers that rear the larvae (27, 55, 56) and food availability in the colony (25). In an exploratory experiment, we additionally found that the temperature at which larvae develop affects the frequency of intercastes (*SI Appendix*, Fig. S1). Thus, to test for plasticity in the static allometry for intercaste traits (Fig. 1 *A* and *B*), we varied caregiver genotype, rearing temperature, and food availability while controlling for larval genotype and other components of the rearing environment.

We measured the body size of the resulting adults, counted their ovarioles, and scored the presence/absence of vestigial eyes. Over 96.5% of *O. biroi* adults of clonal line B had either two or four ovarioles (*SI Appendix*, Fig. S2), and vestigial eyes were either absent or present as small, darkened eye spots (Fig. 1*C*). Static allometries are often used to depict correlations between two or more continuous traits, but they can also be used to show the probability of developing either state of a binary trait (Fig. 1 *A* and *B*), which can be modeled using logistic regression. For these analyses, the estimated probability of exhibiting a state at a given body size reflects the estimated proportion of individuals that display the state at that body size.

**Fig. 2.**
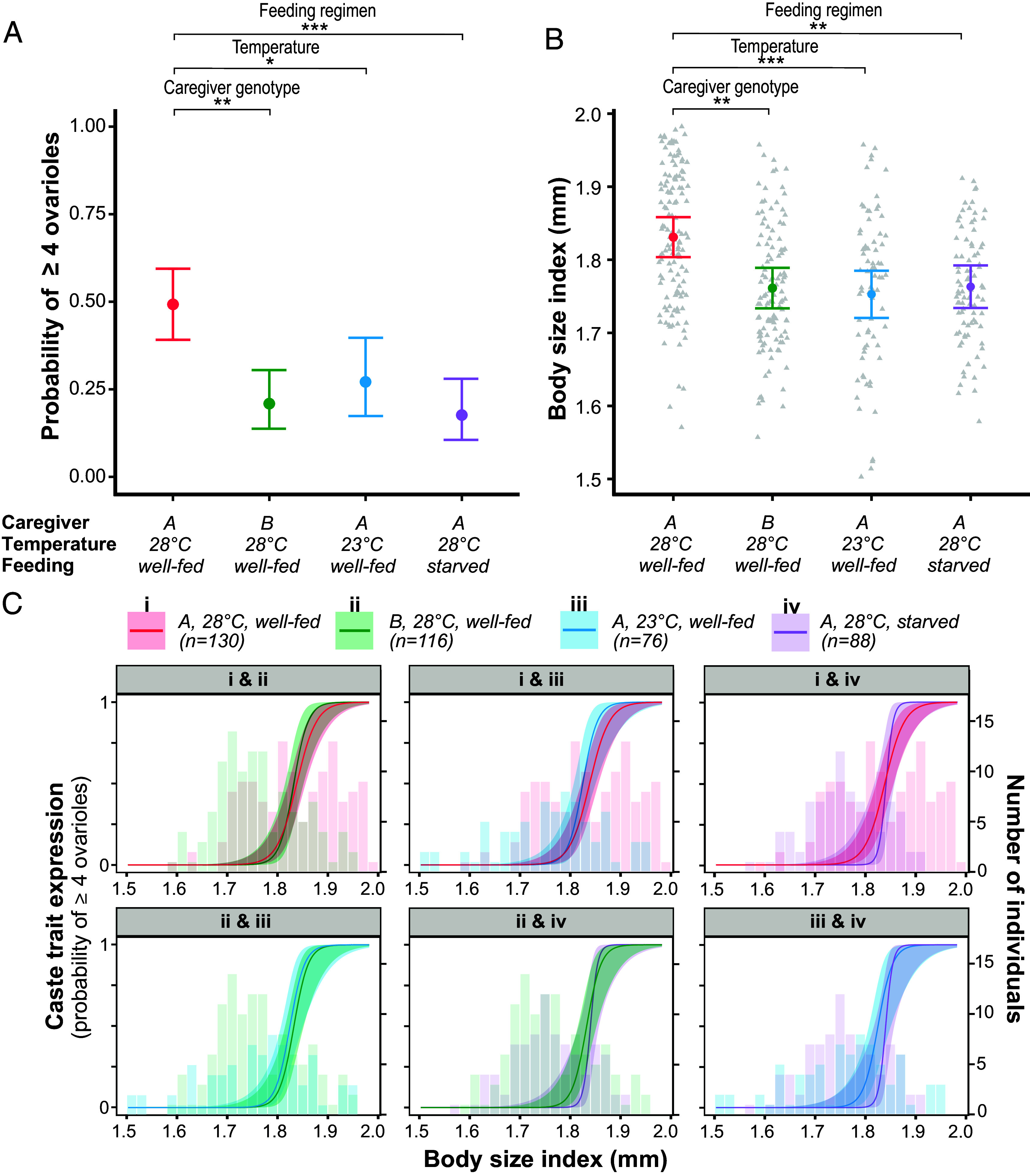
Developmental plasticity in caste trait expression is coupled with body size plasticity, and allometric plasticity is absent. (*A*) Developmental plasticity in ovariole development was induced by manipulating larval rearing environment. All three environmental factors (caregiver genotype, temperature, and food quantity) significantly influenced the probability of developing four or more ovarioles. (*B*) Differences in the body size of reared individuals across experimental conditions reveal body size plasticity. Caregiver genotype, temperature, and feeding regimen influenced the average body size of adults. Each triangle represents an individual ant. (*C*) The estimated static allometries for ovariole development across different larval rearing environments, with all possible pairwise comparisons shown in individual panels. Neither the intercepts nor slopes of the logistic regression differed across conditions, suggesting negligible or no allometric plasticity. Size-matched individuals from different environmental backgrounds exhibit the same caste morphology on average. Although each condition included 250 larvae initially, sample sizes (n) represent the number of individuals measured for each condition, which corresponds to the number of larvae that survived to at least 10 d after adult eclosion. Color-coded histograms represent the size distribution for each condition. Shaded areas and error bars represent 95% C.I.s. All conditions of the experiment reported in this figure were conducted with clonal line B brood. Asterisks denote a significant difference (*P < 0.05; **P < 0.01; ***P <0.001).

Caregiver genotype, temperature, and feeding regimen influenced the probability of developing four or more ovarioles [GLMM, Bonferroni-adjusted *P*-values: caregiver genotype, χ^2^(1) = 14.01, *P* = 0.001; temperature, χ^2^(1) = 6.99, *P* = 0.025; feeding, χ^2^(1) = 16.44, *P* < 0.001; Fig. 2*A*], and vestigial eyes [GLMM, Bonferroni -adjusted *P*-values: genotype, χ^2^(1) = 12.63, *P* = 0.001; temperature, χ^2^(1) = 6.05, *P* = 0.042; feeding, χ^2^(1) = 16.37, *P* < 0.001; see *SI Appendix*, Fig. S3*A*]. Thus, all three experimental manipulations affected caste trait expression. Specifically, larvae had a decreased probability to develop four or more ovarioles and vestigial eyes when reared by line B caregivers, when fed less, and when developing at a lower temperature (Fig. 2*A* and *SI Appendix*, Fig. S3*A*). Only 3 of 127 individuals that developed four or more ovarioles lacked vestigial eyes, and the static allometries for ovariole- and eye development were not significantly different (*SI Appendix*, Fig. S3*B*). Because of the tight correlation between these two traits, we show figures summarizing results for plasticity in eye development only in the supplementary material.

Importantly, while the probability of developing four or more ovarioles differed across experimental conditions [GLMM, condition: χ^2^(3) = 22.51, *P* < 0.0001], condition had no significant effect after controlling for body size [GLMM, unadjusted *P*-values: body size, χ^2^(1) = 47.45, *P* < 0.0001; condition, χ^2^(3) = 1.71, *P* = 0.635]. Accordingly, the average body size of reared adults was influenced by all three environmental factors [GLMM, Bonferroni-adjusted *P*-values: caregiver genotype, χ^2^(1) = 12.39, *P* = 0.001; temperature, χ^2^(1) = 13.21, *P* < 0.001; feeding regimen, χ^2^(1) = 11.14, *P* = 0.003; Fig. 2*B*]. This shows that the differences between rearing conditions in the probability of developing four or more ovarioles is fully predicted by differences in average body size.

To explicitly test whether the static allometry of caste-associated traits can vary across rearing conditions, we applied a binomial GLMM with body size, condition, and an interaction between condition and body size as fixed effects. Here, a generalized linear model with two parameter estimates (slope and intercept) for each condition was generated such that the log of the odds of expressing a trait was a function of body size. Converting the log of odds to a probability value produces a sigmoid-shaped function when plotted against body size (*SI Appendix*, Fig. S4). Neither condition nor the interaction term influenced the probability of developing four or more ovarioles [GLMM, unadjusted *P*-values: condition, χ^2^(3) = 0.90, *P* = 0.826; interaction, χ^2^(3) = 3.27, *P* = 0.352], or vestigial eyes [GLMM, unadjusted *P*-values: condition, χ^2^(3) = 2.40, *P* = 0.493; interaction, χ^2^(3) = 2.26, *P* = 0.519]. This means that neither the intercept nor the slope of the logistic regression for either ovariole or eye development differed significantly across different rearing environments (Fig. 2*C* and *SI Appendix*, Fig. S4). Post hoc tests corroborated that static allometries did not differ significantly between any pair of tested conditions (Fig. 2*C* and *SI Appendix*, Table S1). This implies that allometric plasticity was negligible or absent in this experiment. Since size-matched individuals from different environmental backgrounds did not differ in caste morphology, this suggests that body size plasticity (Fig. 1*A*), rather than allometric plasticity (Fig. 1*B*), underlies developmental plasticity in *O. biroi* caste morphology. In other words, differences in mean caste trait expression across conditions were coupled to shifts in the underlying size distribution of individuals rather than shifts in static allometry (Fig. 2*C*).

### Genotypic Effects On Caste Morphology.

**Fig. 3.**
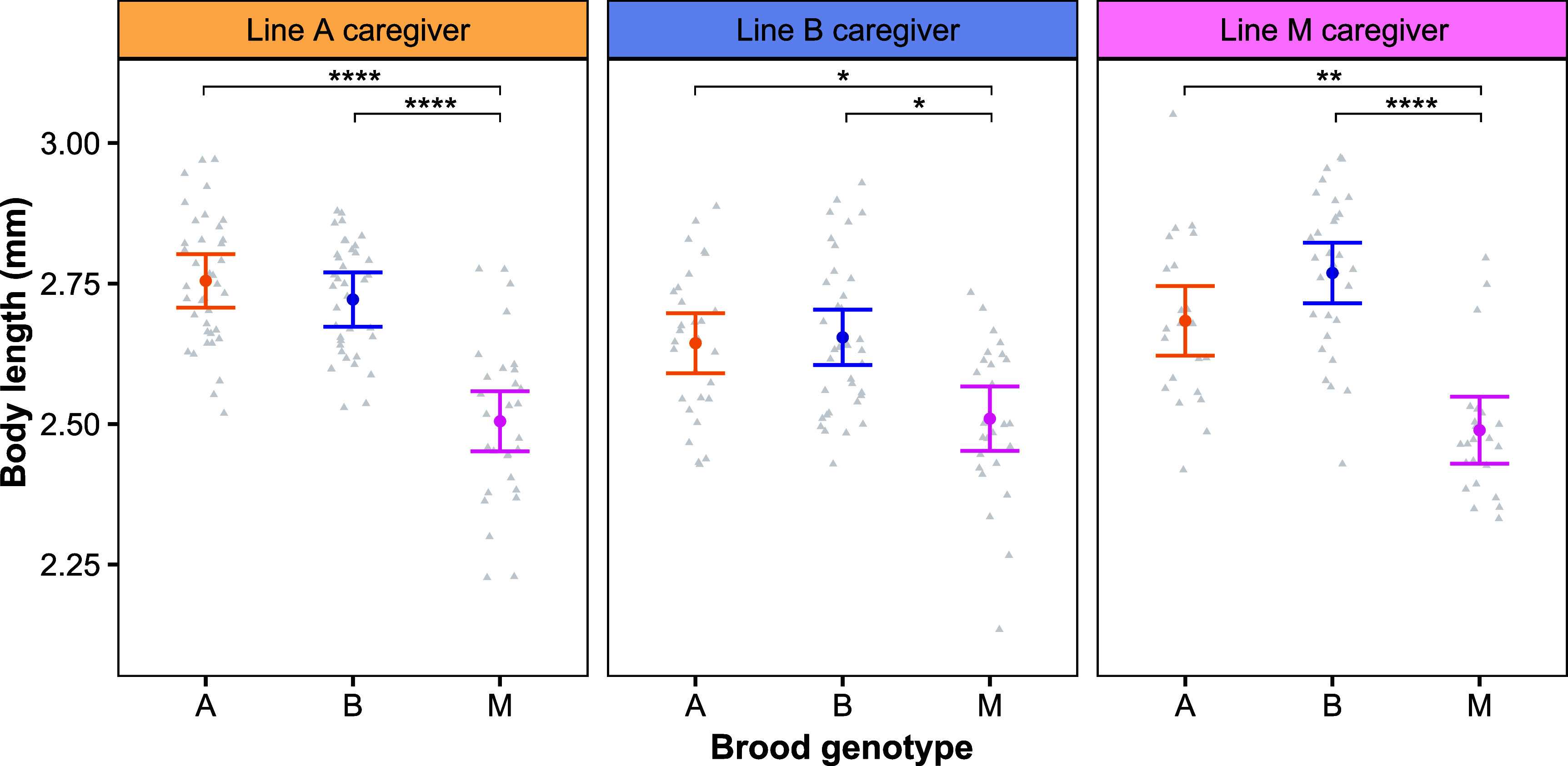
Genotypes differ in average body length in controlled environments. The adult body length attained by brood of three clonal lines (A, B, and M; denoted on the x-axis) reared by caregivers of three different clonal lines. Each panel shows brood of different genotypes reared by the same genotype of caregiver. Brood genotype affected final body length, with line M brood attaining smaller body length on average compared to line A and B brood, regardless of caregiver genotype. Body length was not significantly different between clonal lines A and B. Gray triangles represent individual ants, and error bars represent 95% C.I.s. Body length represents the measured distance from the most anterior part of the head to the posterior end of the gaster in the dorsal view. Asterisks denote a significant difference (*P < 0.05; **P < 0.01; ****P < 0.0001). Data reanalyzed from Jud et al. (55).

We next evaluated the role of genetic variation on caste morphology. In *O. biroi,* larvae from different clonal genotypes, when reared in identical conditions, give rise to adults that differ in average body sizes (55). We reanalyzed published data from a fully factorial cross-fostering experiment (55) using the clonal lines that we included in our experiments (A, B, and M). The body length of focal individuals was influenced by caregiver genotype [LMM: χ^2^(2) = 9.50, *P* = 0.009], brood genotype [LMM: χ^2^(2) = 108.07, *P* < 0.0001], and a genotype–genotype interaction [LMM: χ^2^(4) = 10.24, *P* = 0.037]. Most relevant to the current study, line M brood attained smaller adult body lengths than line A and B brood, regardless of the genotype of the caregivers rearing them [Tukey’s HSD, Line A caregiver: M vs A, *P* < 0.0001; M vs B, *P* < 0.0001; Line B caregiver: M vs A, *P* = 0.036; M vs B, *P* = 0.013; Line M caregiver: M vs A, *P* = 0.002; M vs B, *P* < 0.0001; Fig. 3]. This shows that genetic differences between even closely related clonal lines of *O. biroi* can affect body size under identical environmental conditions.

**Fig. 4.**
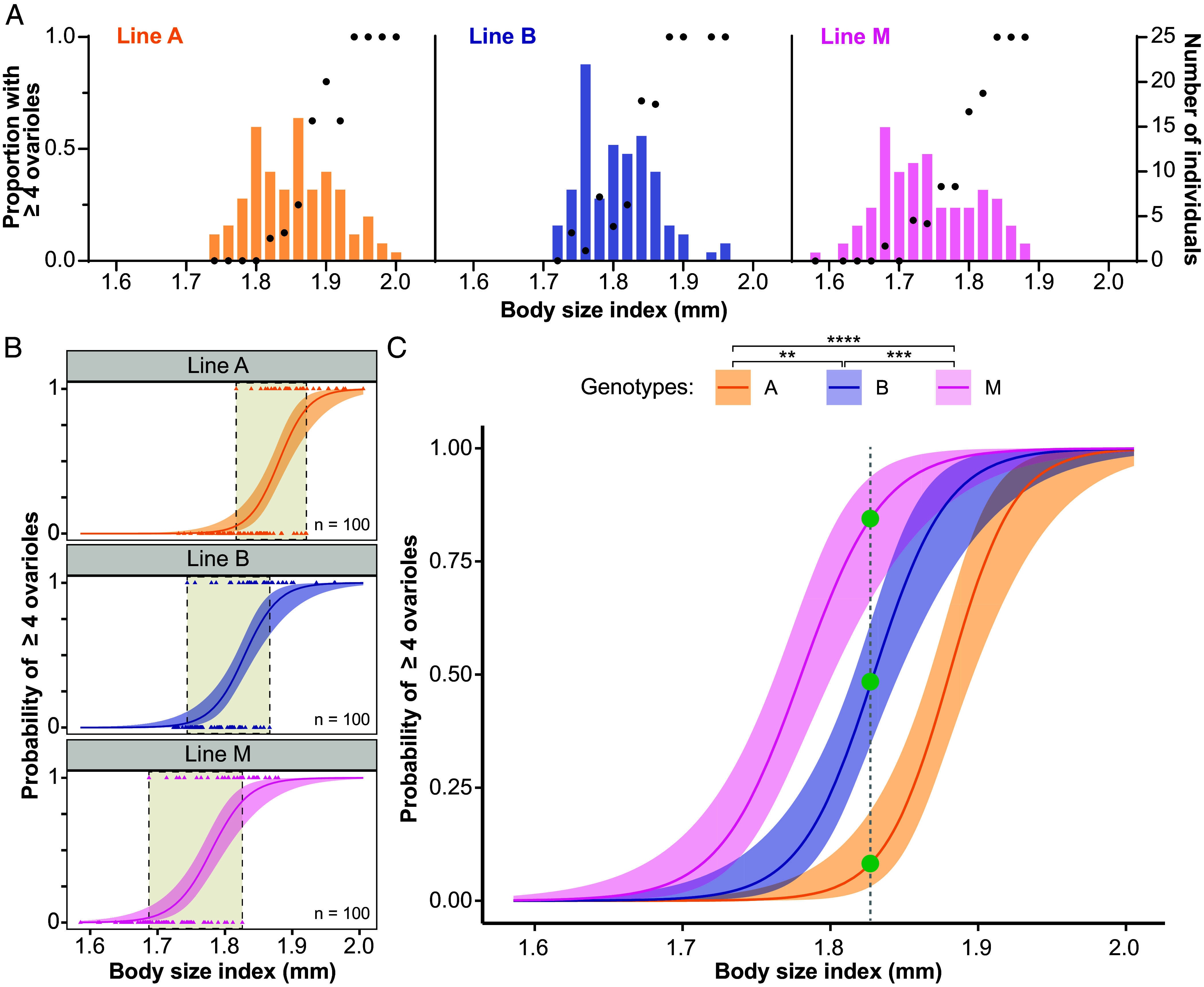
Static allometries for caste-associated traits differ between genotypes. (*A*) Observed proportions of intercastes across bins of a size histogram for each of three clonal genotypes (A, B, and M). (*B*) Estimated static allometries for ovariole development across three genotypes. Each panel shows the static allometry, modeled as a logistic regression function, along with the raw data where triangles represent individual ants (n = 100 per genotype). Shaded boxes represent the size overlap between regular workers and intercastes. (*C*) Same genotype-specific static allometries as shown in (*B*) but overlaid. The relationship between ovariole number and body size differs across genotypes, with line M ants developing four ovarioles at smaller body sizes than lines B and A, and line B ants developing four ovarioles at smaller body sizes than line A (i.e., M > B > A). Size-matched individuals of different genotypes (shown by green circles) differ in caste trait expression (i.e., the probability of developing four or more ovarioles). Areas shaded in color represent 95% C.I.s. Asterisks denote a significant difference in the intercept of the logistic regression function between genotypes (**P < 0.01; ***P <0.001; ****P < 0.0001).

We then conducted an experiment in which we measured individuals from the three different clonal lines across a range of body sizes. We observed that intercaste proportions for some genotypes were higher at smaller body sizes relative to other clonal genotypes (Fig. 4*A*). To test for genotype-specific static allometries, we estimated and compared the allometric relationship between body size and ovariole number in these three clonal lines (Fig. 4*B*). The intercept but not slope [GLM, unadjusted *P*-values: genotype: χ^2^(2) = 59.76, *P* < 0.0001; interaction of body size and genotype, χ^2^(2) = 0.66, *P* = 0.717] of the static allometry for ovariole development differed across genotypes (Fig. 4*C*). Line M developed four or more ovarioles at smaller body sizes than lines A and B (Tukey’s HSD: M vs A, *P* < 0.0001; M vs B, *P* < 0.001), and line B developed four or more ovarioles at smaller body sizes than line A (Tukey’s HSD: B vs A, *P* < 0.01; Fig. 4*C*). Similarly, the intercept but not slope [GLM, unadjusted *P*-values: genotype: χ^2^(2) = 56.16, *P* < 0.0001; interaction of body size and genotype, χ^2^(2) = 0.94, *P* = 0.625] of the static allometry for eye development differed across genotypes (*SI Appendix*, Fig. S5). Line M developed vestigial eyes at smaller body sizes than lines A and B (Tukey’s HSD: M vs A, *P* < 0.0001; M vs B, *P* = 0.011), and line B developed vestigial eyes at smaller body sizes than line A (Tukey’s HSD: B vs A, *P* = 0.008). In other words, size-matched individuals of different genotypes displayed differences in the probability of exhibiting intercaste morphology (Fig. 4*C*).

Comparing the effect of genotype (Fig. 4*C*) and environmental condition (Fig. 2*C*) on caste trait static allometry suggests that the effect of genotype was stronger than the effect of environment. Power analyses indicate that we had high statistical power (≥0.90 in most pairwise comparisons of environmental conditions) to detect environmental effects as large as the observed genotype effect on intercaste development when controlling for body size (*SI Appendix*, Table S2). This supports the conclusion that the environmental variables examined in this study did not influence caste trait static allometry to an equal or greater degree than genetic factors did. Altogether, the results show that closely related wild-type genotypes can vary in the static allometry of caste traits (Fig. 4*C*), but we found no evidence that static allometry varies within genotypes due to rearing environment (Fig. 2*C*).

## Discussion

### Developmental Plasticity in Caste Morphology and Body Size are Coupled.

Experimentally manipulating caregiver genotype, temperature, and food quantity affected caste trait expression and body size in tandem but did not significantly affect the allometric relationship between caste morphology and body size. Thus, we found no evidence for allometric plasticity in response to these environmental variables. Instead, environmentally induced changes in caste trait expression were invariably associated with changes in body size (Fig. 1 *A* and *B*). Our results are thus consistent with the prior hypothesis that ant caste phenotypes can be described by plotting caste trait expression as a function of body size (10–12, 14). While size was highly predictive of caste trait expression, this does not imply that body size is itself the causal factor determining caste phenotype.

As for other insect traits, developmental plasticity that regulates the caste phenotypes of adults may be modular (i.e., semiautonomous), such that tissue and body growth are regulated by distinct environmental variables and/or exhibit differential sensitivity to environmental stimuli (57–59). This would allow for tissue-specific growth that is disproportionate to, or completely independent of, the typical corresponding change in body size. For example, increased temperature could disproportionally increase cell proliferation in the wing or eye-antennal imaginal discs relative to the rest of the body, resulting in wing or eye development at smaller body sizes and thereby altering its allometric relationship with body size (60). Furthermore, modular trait growth would entail that predictor variables other than body size explain variation in trait expression, reducing the coefficient of determination (R^2^) for models that only include body size as an independent variable. In *Drosophila melanogaster*, the static allometry between body size and trait size varies depending on the environmental factor generating body size variation (e.g., rearing density, temperature, and nutrition) (59). This implies that environmental factors regulate tissue growth and body size independently to some degree, indicating modularity in tissue growth (59). Consequently, flies of the same genotype and size differ in trait expression across different rearing environments. In contrast to these studies, we failed to detect variation in the static allometry of caste traits in ants when manipulating three environmental variables that generate body size variation (caregiver genotype, temperature, and food quantity). Environmentally induced changes in caste trait expression strictly coincided with changes in body size, implying limited or no modularity in tissue growth in *O. biroi*. However, further research is needed to determine whether and how broadly these findings apply to other caste traits and ants in general.

This lack of allometric plasticity might be explained if circulating hormones and/or growth factors jointly coordinate tissue growth and body size, causing environmental effects to influence tissue growth and body size in tandem (57, 58). If size and caste phenotypes are systemically regulated in this manner, static allometry should not vary based on the environmental factor generating variation in body size (i.e., the static allometry does not change across environmental conditions). Put differently, the environmental context may affect size and caste trait expression in concert, such that final body size, not environmental context, is always the best predictor of caste morphology. Our finding that body size is highly predictive of caste trait expression [GLMM with body size as the only fixed effect: Nakagawa’s (61) marginal pseudo-R^2^ = 0.91 (0.86, 0.95); correct classification rate (i.e., model accuracy) = 0.93 (0.90, 0.95); area under curve (AUC) of ROC curve = 0.98] is consistent with this model.

The question of whether body size is a fundamental determinant of ant caste morphology, or just one of multiple traits that differ between castes, has been discussed previously (9–12, 41, 62, 63). In some ant species, female castes can overlap in body size, and this has been used to argue that caste trait expression can be decoupled from body size (41). For continuous traits, we previously suggested that, if caste is fundamentally coupled to overall size, the observed overlap can be parsimoniously explained by developmental noise, measurement error, and/or genetic variability in the static allometries among sampled individuals (12). Given that genetic variability in the static allometries for caste traits is widespread, random sampling of individuals in sexual and genetically diverse ant populations may often generate size overlap between castes. In this study of a parthenogenetic ant, we analyzed binary caste-associated traits and used a logistic regression function to estimate genotype-specific static allometries. We observed a considerable overlap in the body sizes of workers and intercastes within genotypes (Fig. 4*B*), yet we found that a probabilistic size-based model was highly predictive of caste. For discrete traits, if a logistic regression function accurately reflects the relationship between body size and the probability of expressing the trait, a certain degree of size overlap can be expected. Thus, for probabilistic categorical traits, an overlap in body size between different morphs may be observed even in the absence of genetic variation.

Treatment of worker-destined larvae with a juvenile hormone analog in *Monomorium pharaonis* increases body size and promotes the expression of queen-like adult morphology, although it does not induce ovarian development (62). In this and other ant lineages with sterile workers, ovarian development might be impossible in worker-destined embryos because they lack germ line primordia (5, 62, 64). Interestingly, even in a species that has evolved a sterile worker caste, all other queen-associated anatomical and morphological traits retain their ancestral association with body size and each other (62). These findings confirm observations from across ants showing that caste phenotypes are tightly coupled with adult body size (10): Even in worker-destined larvae that lack the capacity to develop ovaries, experimental perturbations that increase their body size also induce a correlated suite of queen-like caste traits. However, this does not imply that body size is causal to caste morphology, and future research is required to identify the molecular and developmental mechanisms responsible for the pervasive association of caste phenotypes with each other and overall body size. How body size and caste-associated tissue differentiation are developmentally coupled remains an outstanding question. Either both body size and tissue differentiation are jointly regulated by the same molecular factors (e.g., juvenile hormone), or tissue differentiation is downstream of body size or a correlated factor. In either case, this recent discovery and our results from the clonal raider ant are both consistent with our prior hypothesis that, during caste development, “the formation of adult phenotypes [is] a function of size or factor(s) tightly correlated with size”(12).

### The Genotype Affects both Body Size and Static Allometry.

Our analysis of a previously published dataset (55) shows that larvae of different closely related clonal genotypes (51) raised under identical environmental conditions attain different adult body sizes, demonstrating genetic effects on final body size. Genetic effects on body size, and consequently caste development, are widespread in ants (28, 29, 35, 36). In most cases, strong genetic caste biases (65, 66) presumably arise indirectly due to a genetic effect on adult body size [(9–12); see (9) for a detailed discussion of genetic effects on caste morphology].

We also found that the static allometries for caste traits differ between genotypes, revealing genetic effects on allometry. This is reminiscent of a recently described *O. biroi* caste differentiation mutant that expresses an aberrant suite of queen-like traits at worker-like body sizes (9), as well as evidence for genetic effects on the static allometry of caste traits in *Pheidole* (38, 39) and *Camponotus* (37). Taken together, this raises the possibility that genetic variation in the static allometries for caste-associated traits is widespread in ants. Given that the clonal lines examined here are closely related and are ultimately derived from the same native range (51), this suggests that genetic variation for body size and the static allometries of caste traits are under the purview of natural selection in the populations of most, if not all, ants. Mapping these genetic variants, both within and across ant species, will greatly advance our understanding of caste development and evolution. For instance, such studies may reveal molecular factors that regulate caste morphology independently of body size, as well as the genetic elements that have undergone selection to generate the diversity in static allometries across ants.

If size-matched individuals of different genotypes express different caste morphologies, there must be some genetic factor (or factors) that modifies the relationship between caste trait expression and body size. Genotypic differences in the threshold size for queen-associated tissue differentiation may be a result of genetic variability in the timing or level of secretion of systemic hormones and/or growth factors closely associated with body size or in the responsiveness to them, and/or genetic variability in the initial number of progenitor cells or their inherent rate of proliferation (14, 67). The underlying mechanism generating differences in static allometries across clonal genotypes of *O. biroi* remains an open question. Our data also corroborate previous work showing differences in caregiver–brood interactions across different clonal genotypes (27, 55, 56). *Temnothorax curvispinosus* ants show a negative genetic correlation between direct and indirect (maternal and sibling) effects on caste ratio, suggesting that some genotypes show a reduced probability for queen development in individual larvae while also showing increased adult brood care efficiency that indirectly increases the likelihood that larvae develop into queens (28). Such genetic correlations can occur through either genetic linkage or pleiotropy. The evolutionary dynamics shaping genetic variability in brood care traits and caste development require further study. For example, future research is needed to clarify how intra- and intercolony selection pressures may act on these traits, whether such traits show genetic linkage or pleiotropy, and how this may differ between sexual and asexual ants.

## Conclusions and Outlook

Here, we show that the genotype of an individual can affect both its body size and the allometric relationship between caste morphology and size, implying that natural selection can act on genetic variation affecting body size and/or static allometries to change caste morphology. Genetic variability in static allometries indicates that the genotype can change tissue growth without the corresponding expected effect on body size.

We also found that, within genotypes of the clonal raider ant, developmental plasticity of caste-associated physical traits is coupled to effects on body size. We did not detect any plasticity in the static allometry for caste-associated traits, meaning that size-matched individuals of the same genotype from variable environmental backgrounds do not differ in mean caste trait expression. Although our data cannot exclude the possibility that larger sample sizes may detect subtle allometric plasticity, our results indicate that the allometric relationship between body size and caste-associated traits in *O. biroi* is genetically encoded and largely robust to environmental perturbation. Systemic molecular coordination of tissue growth and body size provides a compelling and parsimonious mechanism to explain our results (11).

Allometric plasticity has been observed for polyphenic traits in other animals, but investigations of this phenomenon remain limited in taxonomic scope. Food quality affects the static allometry for male traits in dung beetles and mites (40, 43, 44), as does temperature in the dung beetle *Onthophagus taurus* (45). In ants, however, the few studies reporting environmental or experimental effects on allometric scaling either included genetic differences between conditions as a confounding factor (68–70) or compared static allometries between groups of individuals that differed in body size (71). If a static allometry is nonlinear, differences in scaling relationships observed across nonoverlapping size ranges may simply represent different segments of a curved reaction norm, rather than genuine differences in the mechanisms of allometric scaling. By comparing the static allometry of groups that span the same size range, one can unambiguously determine whether environmental conditions have altered the scaling relationship itself. Put differently, perturbations that affect the size distribution of a population may also change the position of that population on the reaction norm. Consequently, to test whether the reaction norm itself differs between treatments, we compared populations with overlapping size distributions to see whether phenotypes are the same or different along the same interval of the reaction norm.

Further controlled studies of developmental plasticity across numerous ant species are needed to test whether the absence of allometric plasticity we observed in *O. biroi* generalizes to other ants. If allometric plasticity is present, then it is likely that the degree of plasticity is variable across genotypes, and selection could act on this genetic variation to shape caste morphology. It would also imply that environmental variables can decouple caste morphology and body size due to independent regulation of tissue growth and body size. On the other hand, if allometric plasticity is limited or absent in ants, it poses questions about why and how ant caste traits differ in this regard compared to other animal traits. While many animal traits are presumably independently regulated by environmental factors to some degree (e.g., 40, 59), it appears that caste traits in ants do not follow this pattern and are distinctly more integrated. This may be a result of selection favoring a tight coupling of developmental plasticity between the caste-associated traits that distinguish the queens and workers of most ant species.

## Materials and Methods

### Experimental Design and Genotypes Used.

Lines A and B belong to a clade of genotypes native to Bangladesh, but they have successfully invaded other regions (51). Line M is currently only known from Bangladesh, where stock colonies were originally collected (51). Our stock colonies of line A were collected on Okinawa, Japan, and those of line B were collected on St. Croix, U.S. Virgin Islands (52).

In the first experiment, we collected recently eclosed callows from stock colonies of line A (C16) and line B (STC6). We ensured that line A and line B caregivers were age-matched by simultaneously setting up ten colonies composed of 30 line B callow workers each and 30 colonies with 30 line A callow workers each. These colonies were housed in Petri dishes with a plaster of Paris floor and maintained at 25 °C ± 1 °C and ≥60% relative humidity in a climate-controlled room. Colonies were permitted to lay eggs, and when larvae hatched, the colonies entered the brood care phase. On the day that the last larvae hatched, all larvae were removed from all 40 experimental colonies. To account for the fact that some of the initially 30 workers had died in some of the colonies at this point, all colonies were adjusted to contain 25 adult caregivers. Then, 25 age-matched first instar larvae from a single stock colony of line B (STC6) were swapped into each experimental colony. This way, we were able to control not only for brood genotype and developmental stage but also for putative embryonic maternal effects. Following this setup, we began experimental manipulations of temperature and feeding regimen. The ten colonies with line B caregivers were maintained at 28 °C and fed every 48 h with 16 frozen worker pupae and/or larvae of *Solenopsis invicta* (i.e., well fed status). Ten colonies with line A caregivers were maintained at 28 °C and well fed, another ten were also well fed but maintained at 23 °C, and ten colonies were maintained at 28 °C but relatively starved (i.e., fed every 96 h with only eight frozen worker pupae and/or larvae of *S. invicta*). Thus, there were four conditions that varied either by caregiver genotype, temperature, or feeding regimen, allowing us to test the effect of each environmental factor on the probability of intercaste development, body size, and the allometric relationship between caste morphology and body size. Larvae of *O. biroi* inhibit the ovaries of caregiving adults (25, 72), ensuring that the young adults of our focal cohorts were exclusively derived from the cross-fostered line B larvae. Consistent with this, we never observed eggs in experimental colonies during our daily checks after line B focal larvae had been provided to adults.

To address whether clonal lines differ in average body size, we utilized a previously published dataset that measured the body length of adults that were reared under controlled experimental conditions (48). We reanalyzed the data available for lines A, B, and M, since these were the genotypes that we had available for further experimentation. Then, to estimate the trait-to-body-size allometries for the three clonal genotypes, we measured body size, counted ovarioles, and scored eye development for 100 ants from a stock colony of line A (C16), line B (STC14), and line M (BG14). For each stock colony, 40 ants were haphazardly collected and only the ten largest ants (measured qualitatively) were sampled. This process was repeated ten times to have 100 total ants sampled per stock colony. This sampling strategy enriches for intercastes (which typically compose <5% of the colony) and large ants in the range of size overlap between regular workers and intercastes.

### Body Size and Morphology.

We first estimated the full body length of 20 individuals by summing the length of the head, thorax, petiole, postpetiole, and first gastral segment from dorsal view images. We then compared these body length measurements to a body size index, which is the full body length minus the length of petiole and postpetiole (*SI Appendix*, Fig. S6*A*). This body size index was highly correlated with full body length measurements (*p* < 0.0001, R^2^ = 0.99; see *SI Appendix*, Fig. S6*B*). Thus, for all subsequent ants, we measured and summed the length of the head, thorax, and first gastral segment to obtain a body size index to be used for statistical analyses. Repeating body size index measurements for 20 individuals confirmed that measurements were highly repeatable (*P* < 0.0001, R^2^ = 0.99; see *SI Appendix*, Fig. S6*C*). Images were taken with a Leica Z16 APO microscope equipped with a Leica DFC450 camera using the Leica Application Suite version 4.12.0 software (Leica Microsystems, Switzerland). Lengths of body segments were measured using ImageJ (73).

Ant ovaries were dissected to determine ovariole numbers. In this study, we treated ovariole numbers and vestigial eyes as binary traits (Fig. 1*C* and *SI Appendix*, Fig. S2) and used a logistic regression function to approximate their allometric relationship with body size. Wing buds do rarely occur in the largest intercastes, but due to limited numbers of ants showing this phenotype, we did not examine this trait. In other systems, caste-associated traits can be treated as continuous and fitted with nonlinear functions (e.g. four-parameter logistic equation, Gompertz equation, etc.) to approximate the static allometry. All raw data for this study, including body size, ovariole number, and eye development scores of all individuals sampled, are available here ((74); Datasets S1–S3).

### Statistical Analyses.

To test the effect of caregiver genotype, temperature, and feeding regimen on probability of developing intercaste morphology, we used a binomial generalized linear mixed model (GLMM) with a logit link function. Caregiver genotype, temperature, and feeding regimen were treated as fixed factors, and colony was treated as a random factor. To test the effect of these same fixed factors on body size, we used a linear mixed model (LMM) with colony as a random factor. Because we tested three different environmental factors, we adjusted *P*-values for multiple comparisons using Bonferroni corrections. To test differences in the intercept and/or slope of the static allometry for ovary and eye development across the four experimental conditions, we used a binomial GLMM with a logit link function with body size, condition, and their interaction as fixed effects, with colony as a random factor. Here, Bonferroni corrections were not applied to be more permissive in detecting potential environmental effects on caste trait static allometry. Significance of the model’s condition and interaction term would indicate significant differences in the intercept and slope of the logistic regression between conditions, respectively.

To examine whether genotypes differ in average body size under identical rearing environments, we applied an LMM with caregiver genotype, brood genotype, and the interaction of genotypes as fixed factors, and colony as a random factor. To test differences in the static allometry of caste-associated traits for different genotypes, we used a binomial generalized linear model (GLM) with body size, genotype, and their interaction as model terms. Post hoc Tukey’s tests comparing genotypes in their average body sizes and static allometries were performed using the package *emmeans*. For all analyses, model assumptions were tested using the function *simulateResiduals* from the *DHARMa* package and significance of model terms was determined using the *Anova* function of the *car* package.

To estimate the pseudo-R^2^ of our logistic regression function representing the allometric relationship between caste morphology and body size of line B, we pooled all 410 individuals sampled in the first experiment and applied a GLMM with only body size as a fixed factor and colony as a random factor. Then we used the *r2_nakagawa* function (75) of the package *performance* to estimate the marginal pseudo-R^2^ of the GLMM. We also calculated the model’s percentage correct classification by creating a confusion matrix using the package *caret*, and area under the receiver operating characteristic curve (i.e., AUC of the ROC) using the package *pROC*, to further evaluate how well body size predicts intercaste morphology. Methods for supplementary experiments are described in the supplementary figure captions found in the *SI Appendix*. Methods for power analyses are detailed in the *SI Appendix*. R codes used for statistical analyses are available on the Dryad Digital Repository (https://doi.org/10.5061/dryad.0p2ngf2c8).

## Supplementary Material

Appendix 01 (PDF)

Dataset S01 (XLSX)

Dataset S02 (XLSX)

Dataset S03 (XLSX)

## Data Availability

Raw data; R code for statistical analyses; input files for statistical analyses data have been deposited in Dryad (https://doi.org/10.5061/dryad.0p2ngf2c8) (74). All study data are included in the article and/or supporting information. Previously published data were used for this work [This manuscript includes a reanalysis of previously published data from the following study (55). No special permission or copyright is required to reuse these data, and the source study is properly cited in the current manuscript.].
