# Supplementary material for: Static allometries of caste-associated traits vary with genotype but not environment in the clonal raider ant
Source: Proc Natl Acad Sci U S A. 2025 Jul 22;122(30):e2501716122. doi: 10.1073/pnas.2501716122 (PMC12318203; doi:10.1073/pnas.2501716122)
Supplement: Supplementary file 1 — Appendix 01 (PDF) [file pnas.2501716122.sapp.pdf]

PNAS

Supporting Information for

Static allometries of caste-associated traits vary with genotype but not environment in the clonal raider ant

Patrick K. Piekarski*[1], Stephany Valdés-Rodríguez[1,2], Waring Trible[3], and Daniel J.C. Kronauer*[1,2]

[1]Laboratory of Social Evolution and Behavior, The Rockefeller University, New York, NY 10065, USA

[2]Howard Hughes Medical Institute, New York, NY 10065, USA

[3]John Harvard Distinguished Science Fellowship Program, Harvard University, 52 Oxford Street, Cambridge, MA 02138, USA

*Authors for correspondence: pkpiekarski@gmail.com; dkronauer@rockefeller.edu

This PDF file includes:

- Extended Materials and Methods
- Figures S1 to S6
- Tables S1 and S2
- Legends for Datasets S1 to S3

Other supporting materials for this manuscript include the following:

- Datasets S1 to S3
- R code for statistical analyses
- CSV files S1 to S3

Data is available from the Dryad Digital Repository: https://doi.org/10.5061/dryad.0p2ngf2c8

Extended Materials and Methods

Due to logistical reasons, we did not perform a full-factorial experiment including all combinations of genotype and environmental factors, which would permit quantification of the relative contributions of genetic versus environmental effects on the static allometry between body size and other caste-associated traits. Instead, we conducted experiments that separately evaluated whether there was any evidence that either genotype or environment could affect the static allometry between size and other caste-associated traits. In the first experiment, we tested whether three different environmental factors (caregiver genotype, temperature, and feeding regimen) induced allometric plasticity at all – it did not. This result informed our experimental design for the subsequent experiment where we tested whether genotype affected static allometry – it did. In other words, our study involves two separate experiments, each explicitly asking a different question: (Experiment 1) Do caste traits of *O. biroi* exhibit allometric plasticity? (Experiment 2) Is there genetic variation in the static allometry of caste traits?

Although our study design cannot quantify the exact relative contribution of genotype and environmental factors on static allometry, it is possible to address the following question: if environment influences caste trait expression (controlling for size) with an effect size comparable to that observed due to genotype, how likely were we to detect it? To assess the statistical power of our first experiment to detect an environmental effect on caste trait static allometry equal to or greater than the genotype effect observed in the second experiment, we performed a simulation-based power analysis.

For this analysis, we first estimated effect sizes for differences among genotypes by fitting a binomial generalized linear model (GLM), where the binary outcome (i.e., intercaste or not) was modeled as a function of body size and genotype. The effect of genotype B relative to genotype A yields an effect on the log-odds scale of approximately 2.078 (i.e., the difference in coefficient estimates for genotype B relative to genotype A). A GLM was then applied to all the data from experiment one, using body size as the only fixed predictor. This provided estimates for the intercept (B1 = –110.79) and slope (m = 60.38) parameters of the static allometry for genotype B callows (i.e., logistic regression of y = mx + B1). We then assumed a true environmental effect size of the same magnitude as obtained from the first GLM (i.e., the genotype effect size, B2 = 2.078).

Using the above parameter estimates we then conducted a simulation-based power analysis. First, the two conditions being compared were defined with the sample sizes and mean body sizes observed in our experiment (e.g., for condition 1 vs. 2: n = 130 and 116; while mean size was 1.833 mm and 1.761 mm, respectively). We assumed a standard deviation of 0.09 mm, derived from estimates based on all the body size data. Then, 10,000 datasets were simulated for each pairwise comparison according to the sample sizes and observed mean body size. For each simulated dataset, body sizes were randomly drawn from normal distributions with the specified means and a standard deviation of 0.09 mm. The log-odds of intercaste development were computed for each sampled hypothetical individual in the simulation by summing the intercept, the product of the body size effect and the individual's body size, and, for individuals in one of the conditions, an added effect equal to the genotype effect. The added effect was either a positive value (representing a static allometry shifting left) or a negative value (representing a static allometry shifting right). The log-odds value for each individual was then transformed into a probability using the logistic function, and a binary outcome (i.e., intercaste or not) was simulated based on these probabilities. Then, a binomial GLM was fit to each simulated dataset, and to test

for a significant effect of condition, a likelihood ratio test was performed using the *Anova* function of the *car* package in R. The p-value associated with the condition effect was extracted from these tests. Repeating this simulation 10,000 times yielded a distribution of p-values, and statistical power was estimated as the proportion of simulations with a p-value less than 0.05. Finally, an exact binomial test (Clopper-Pearson method) was applied to these simulation outcomes to derive a 95% confidence interval for the estimated power (see **Table S2**).

We also performed the same statistical approach using our real data to verify whether environmental factors significantly influenced the static allometry between size and ovariole development. This involved looking at each pairwise comparison of conditions individually by fitting a binomial GLM with condition and body size as fixed effects and then performing a likelihood ratio test (**Table S2**). R codes to perform these power analyses are available from the Dryad Digital Repository (https://doi.org/10.5061/dryad.0p2ngf2c8).

Supporting Figures

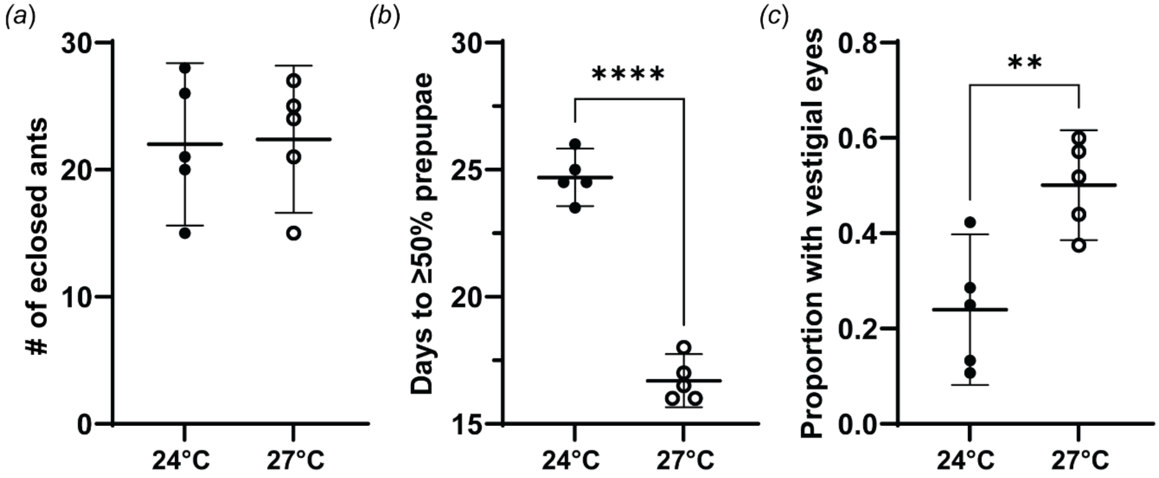

Fig. S1. *Effects of temperature on brood survival, length of larval development, and eye development.* At the beginning of the experiment, all ten colonies consisted of 25 regular workers of clonal line A (C16) and 50 first-instar larvae of clonal line B (STC6). Five colonies were maintained at 24°C for the entire brood care phase, and five at 27°C. All colonies were fed every 48 hours with frozen *Solenopsis invicta* brood. (**a**) Temperature had no effect on the number of brood that eclosed as adults (unpaired t-test: $t(8) = 0.129$, $p = 0.901$). (**b**) Temperature influenced the length of larval development (unpaired t-test: $t(8) = 14.49$, $p < 0.0001$). Larvae reared at 24°C took 8.00 ± 0.55 (mean ± SEM) days longer to reach the prepupal stage compared to larvae reared at 27°C. (**c**) Rearing temperature influenced the proportion of ants that developed vestigial eyes (unpaired t-test: $t(8) = 3.704$, $p = 0.006$). The proportion of individuals that developed vestigial eyes at 27°C was 0.261 ± 0.071 (mean ± SEM) higher compared to larvae reared at 24°C. Each point represents a replicate colony (n = 5 for each treatment); bars represent means and 95% CIs; significant differences are denoted by asterisks (* $p < 0.05$; ** $p < 0.01$; *** $p < 0.001$; **** $p < 0.0001$).

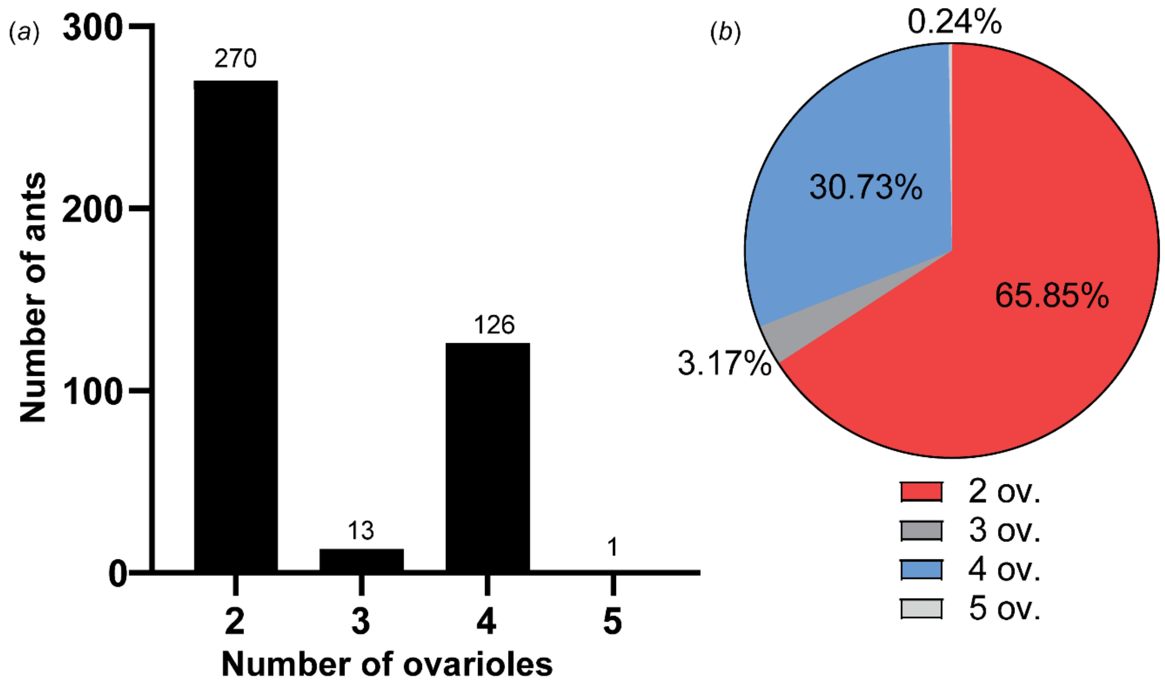

Fig. S2. *Ovariole number variation in Ooceraea biroi (line B) is approximately a binary discrete trait*. (**a**) A frequency distribution of ovariole numbers shows that most individuals have either two or four ovarioles (n = 410). (**b**) Only 3.17% of ants had three ovarioles, while 96.58% of ants had either two or four ovarioles. Thus, in this study we treat number of ovarioles as a binary discrete trait (≥ four ovarioles = 1; ≤ four ovarioles = 0) and use logistic regression models to approximate the static allometry across different genotypes and environments.

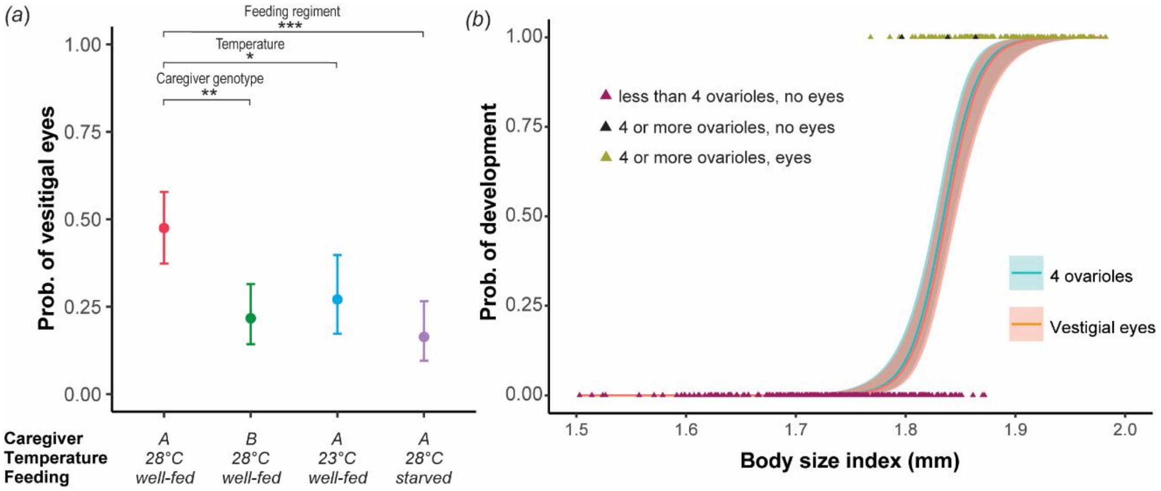

Fig. S3. *Development of vestigial eyes across rearing environments and overlapping static allometries for ovary and eye development.* (**a**) Probability of clonal line B larvae developing into adults with vestigial eyes across four rearing conditions. Caregiver genotype (A vs. B), temperature (23°C vs. 28°C), and feeding regiment (well-fed vs. starved) influenced the probability of developing vestigial eyes (GLMM, Bonferroni-adjusted p-values: genotype, $\chi^2(1) = 12.63$, $p = 0.001$; temperature, $\chi^2(1) = 6.05$, $p = 0.042$; feeding, $\chi^2(1) = 16.37$, $p < 0.001$). Asterisks denote a significant difference (* $p < 0.05$; ** $p < 0.01$; *** $p < 0.001$). (**b**) Allometric relationships for the development of vestigial eyes and four ovarioles overlap, suggesting that the static allometry of these traits with body size is similar in clonal line B. Each triangle represents an individual ant. Out of a total of 410 individuals, 283 individuals had less than four ovarioles and lacked vestigial eyes (red triangles), 124 individuals had vestigial eyes and four or more ovarioles (green triangles), and only 3 individuals had four or more ovarioles and lacked vestigial eyes (black triangles). Bars and shaded areas represent 95% CIs.

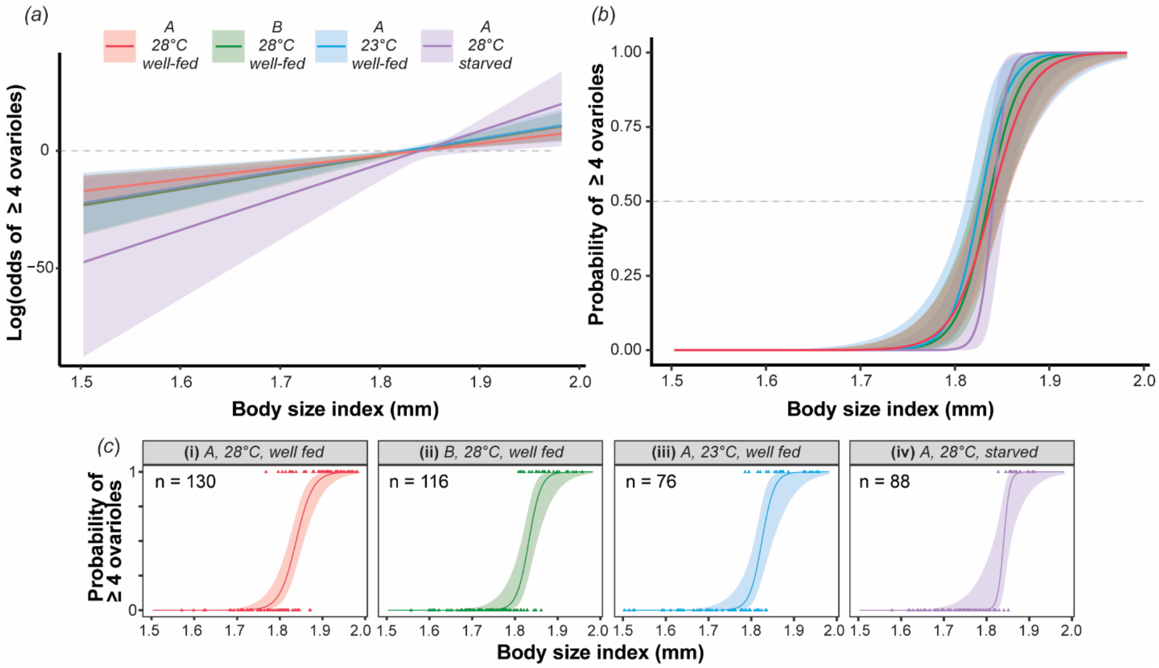

Fig. S4. *Static allometries for ovariole number across different larval rearing conditions estimated by logistic regression.* (**a**) Plots of the binomial GLMM, with colony identity as a random effect and body size, condition and their interaction as fixed effects, showing the log of the odds of expressing ≥ four ovarioles as a function of body size across four different conditions. Neither the intercepts nor slopes of the estimated static allometries significantly differed across conditions. (**b**) Converting the log of odds to a probability produces a sigmoid-shaped function. These logistic regression curves represent the linear functions shown in panel **a**. (**c**) Same allometric functions as shown in panel b but depicted individually; triangles represent individual ants. For all panels, shaded areas represent 95% C.I.s. All conditions of the experiment reported in this figure were conducted with clonal line B brood. All pairwise comparisons of static allometries across conditions are illustrated in **Fig. 2c** of the main text.

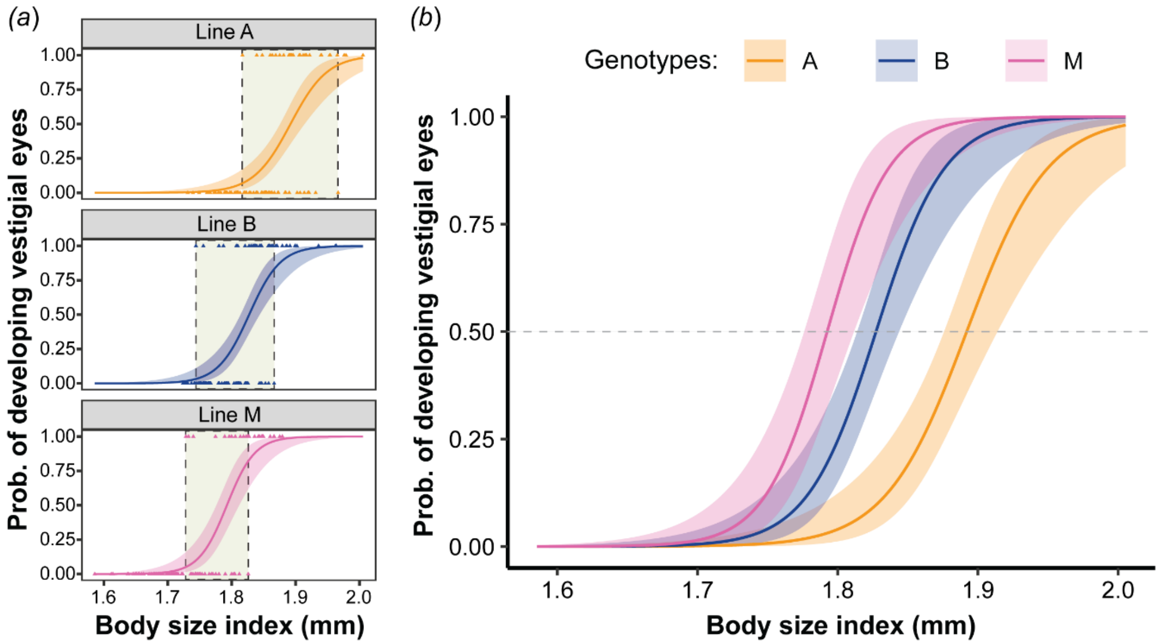

Fig. S5. *Static allometry for vestigial eye development across genotypes.* (**a**) Each panel shows the static allometry for eye development for a given genotype; each triangle represents an individual ant; shaded boxes represent the range of size overlap between ants with and without vestigial eyes. (**b**) Same logistic curves as shown in (**a**) but overlaid. The intercept (GLM, genotype: $\chi^2 = 56.16$, d.f. = 2, $p < 0.0001$), but not slope (GLM, interaction of body size and genotype: $\chi^2 = 0.94$, d.f. = 2, $p = 0.625$), of the static allometry for eye development differed across genotypes. Line M developed vestigial eyes at smaller body sizes than lines A and B (Tukey's HSD: M vs A, $p < 0.0001$; M vs B, $p < 0.05$), and line B developed vestigial eyes at smaller body sizes than line A (Tukey's HSD: B vs A, $p < 0.01$). Shaded areas represent 95% CIs.

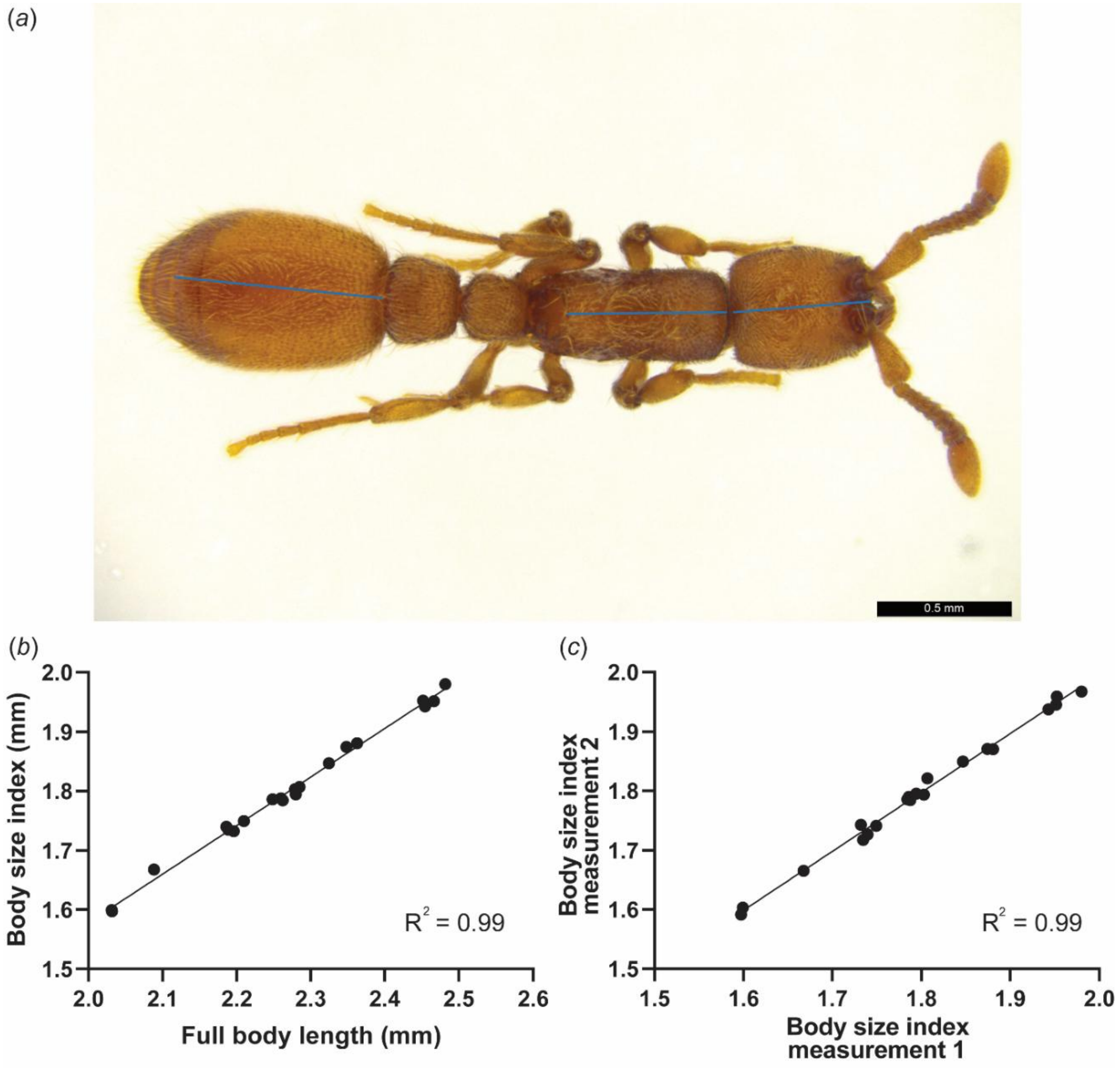

Fig. S6. *Measuring the body size index.* (**a**) The body size index represents the summed length of the head, thorax and first tergum of the gaster (shown by blue lines). (**b**) Full body length measurements attained by summing the length of the head, thorax, petiole, postpetiole and first tergum of the gaster are strongly correlated with body size index (n = 20, $p < 0.0001$, $R^2 = 0.99$). (**c**) Measurements of the body size index were highly repeatable (n = 20, $p < 0.0001$, $R^2 = 0.99$).

Table S1. Post hoc pairwise comparisons of static allometries across all conditions. Analyses were performed using a binomial generalized linear mixed model (GLMM) with two model formulations: (A) one including interaction terms (GLMM: Caste ~ Condition * Body size + (1|Colony)), and (B) one without interactions (GLMM: Caste ~ Condition + Body size + (1|Colony)). For each comparison, unadjusted and Tukey-adjusted p-values obtained from *emmeans* are reported. We did not detect a significant difference between any pair of conditions, even without adjusting for type I error rates. Related to Fig. 2c of main text.

Model	A, 28°C, well-fed vs. B, 28°C, well-fed **(i vs. ii)**		A, 28°C, well-fed vs. A, 23°C, well-fed **(i vs. iii)**		A, 28°C, well-fed vs. A, 28°C, starved **(i vs. iv)**		B, 28°C, well-fed vs. A, 23°C, well-fed **(ii vs. iii)**		B, 28°C, well-fed vs. A, 28°C, starved **(ii vs. iv)**		A, 23°C, well-fed vs. A, 28°C, starved **(iii vs. iv)**	
	p	***p.adj***	***p***	***p.adj***	***p***	***p.adj***	***p***	***p.adj***	***p***	***p.adj***	***p***	***p.adj***
**GLMM A**	0.657	0.971	0.269	0.687	0.853	0.998	0.519	0.917	0.572	0.942	0.240	0.642
**GLMM B**	0.589	0.949	0.972	1	0.142	0.458	0.664	0.973	0.206	0.585	0.152	0.478

Table S2. Statistical power to detect the effect of rearing condition on intercaste probability when controlling for body size and assuming an effect size equal to that obtained for genotype. In this analysis, the magnitude of the genotype effect corresponds to the difference in coefficient estimates for genotypes A and B based on the real data, which was 2.078 on the log-odds scale (i.e., odds ratio of 7.99). The magnitude of the effect comparing genotypes B and M was similar (2.007 on the log-odds scale), but between genotypes A and M it was larger, at 4.084 on the log-odds scale (i.e., odds ratio of 59.40). The mean power to detect an effect size of 2.078 for each pairwise comparison of conditions is in blue text. Since the effect could either decrease or increase the body size threshold for intercaste development (i.e., left- and right-shifting logistic regressions, respectively), power was estimated for both hypothetical scenarios. When performing equivalent statistical tests for each pairwise comparison of conditions, none of the six individual tests recovered a significant effect of environmental condition on the probability of intercaste development. Our power to detect an effect of environmental condition as large as that due to genotype was high (≥0.90 in most cases), meaning we can be confident that rearing environment did not affect the scaling relationship between body size and intercaste development to the same or a larger degree as genotype did.

**Condition**	**i vs ii**		**i vs iii**		**i vs iv**		**ii vs iii**		**ii vs iv**		**iii vs iv**	
Sample size	n = 130, 116		n = 130, 76		n = 130, 88		n=116, 76		n = 116, 88		n = 76, 88	
Mean size (mm)	1.833	1.761	1.833	1.760	1.833	1.761	1.761	1.760	1.761	1.761	1.760	1.761
Effect direction	Left (+)	Right (-)	Left (+)	Right (-)	Left (+)	Right (-)	Left (+)	Right (-)	Left (+)	Right (-)	Left (+)	Right (-)
Power, mean	0.981	0.950	0.937	0.881	0.959	0.908	0.912	0.830	0.938	0.860	0.882	0.785
95% CIs	[0.977, 0.983]	[0.945, 0.954]	[0.932, 0.941]	[0.875, 0.887]	[0.955, 0.963]	[0.903, 0.914]	[0.906, 0.917]	[0.822, 0.837]	[0.933, 0.942]	[0.853, 0.867]	[0.876, 0.888]	[0.776, 0.793]
Type II rate	0.019	0.050	0.063	0.119	0.041	0.092	0.088	0.170	0.062	0.140	0.118	0.215
Real data, condition LRT	$\chi2 = 0.308$, $p = 0.579$		$\chi2 = 0.886$, $p = 0.347$		$\chi2 = 0.075$, $p = 0.784$		$\chi2 = 0.243$, $p = 0.622$		$\chi2 = 0.929$, $p = 0.335$		$\chi2 = 2.391$, $p = 0.122$	

Dataset S1 (separate file). Test of plasticity in the static allometry of caste traits. Sheet 1 contains body size measurements and scores for ovariole and vestigial eye morphology for sampled individuals of clonal line B reared across various environmental backgrounds, which varied in caregiver genotype, temperature, and food availability. Sheet 2 includes raw body size measurements of individual body segments (head, thorax, petiole, gaster) for all ants. Sheet 3 includes additional data on colony level traits, such as number of days it took for >50% of larvae in the colony to become prepupae, number of callows eclosed per colony, and intercaste proportions.

Dataset S2 (separate file). Test of genotypic effects on body size. Set of previously published data (Jud *et al.,* 2022), which includes the measured body lengths of focal individuals of three different clonal genotypes (A, B and M) reared in identical conditions.

Dataset S3 (separate file). Test of genotypic effects on the static allometry of caste traits. Dataset for body size measurements and scores for ovariole and vestigial eye morphology for sampled individuals from three different clonal genotypes (A, B, and M).

R code for statistical analyses (separate file). R file, outlining statistical tests employed in this study (Piekarski2025_PNAS.R). This R code contains all statistical analyses referenced in the main text, as well as power analyses.

CSV file S1 (separate file). Input file for statistical analyses related to the experiment testing for allometric plasticity. This metadata file, for use in R, contains each sample's colony ID, body size index, ovariole score, eye score, and assigned experimental condition.

CSV file S2 (separate file). Input file for statistical analyses related to testing for genotypic differences in body size in a controlled environment. This dataset is from Jud et al. (2022). Dataset for body size measurements. CSV meta file for use in R, containing each sample's colony identification, body length and assigned experimental condition.

CSV file S3 (separate file). Input file for statistical analyses related to genotypic differences in static allometry. This metadata file, formatted for use in R, contains each sample's genotype, body size index, ovariole score, and eye score.
